# Application of PET Tracers in Molecular Imaging for Breast Cancer

**DOI:** 10.1007/s11912-020-00940-9

**Published:** 2020-07-06

**Authors:** Jorianne Boers, Erik F. J. de Vries, Andor W. J. M. Glaudemans, Geke A. P. Hospers, Carolina P. Schröder

**Affiliations:** 1 Department of Medical Oncology, University of Groningen, University Medical Center Groningen, Hanzeplein 1, 9700 RB Groningen, The Netherlands; 2 Medical Imaging Center, Department of Nuclear Medicine and Molecular Imaging, University of Groningen, University Medical Center Groningen, Groningen, The Netherlands

**Keywords:** Breast cancer, Molecular imaging, Positron emission tomography, Technical validation, Clinical validation, Clinical utility

## Abstract

**Purpose of Review:**

Molecular imaging with positron emission tomography (PET) is a powerful tool to visualize breast cancer characteristics. Nonetheless, implementation of PET imaging into cancer care is challenging, and essential steps have been outlined in the international “imaging biomarker roadmap.” In this review, we identify hurdles and provide recommendations for implementation of PET biomarkers in breast cancer care, focusing on the PET tracers 2-[^18^F]-fluoro-2-deoxyglucose ([^18^F]-FDG), sodium [^18^F]-fluoride ([^18^F]-NaF), 16α-[^18^F]-fluoroestradiol ([^18^F]-FES), and [^89^Zr]-trastuzumab.

**Recent Findings:**

Technical validity of [^18^F]-FDG, [^18^F]-NaF, and [^18^F]-FES is established and supported by international guidelines. However, support for clinical validity and utility is still pending for these PET tracers in breast cancer, due to variable endpoints and procedures in clinical studies.

**Summary:**

Assessment of clinical validity and utility is essential towards implementation; however, these steps are still lacking for PET biomarkers in breast cancer. This could be solved by adding PET biomarkers to randomized trials, development of imaging data warehouses, and harmonization of endpoints and procedures.

## Introduction

Over the last decade, there has been an increasing interest in molecular imaging with positron emission tomography (PET), in particular in the field of oncology. PET imaging is a noninvasive tool to obtain qualitative and quantitative whole-body information of biological processes. Molecular imaging in breast cancer (BC) is of particular interest, as it can visualize the estrogen receptor (ER), human epidermal growth factor receptor 2 (HER2), and proliferation. However, molecular imaging with PET has not been widely adopted in clinical practice of BC. Only two radiotracers (2-[^18^F]-fluoro-2-deoxyglucose ([^18^F]-FDG) and sodium [^18^F]-fluoride ([^18^F]-NaF)) are incorporated in cancer management guidelines, such as National Comprehensive Cancer Network (NCCN) and European Society for Medical Oncology (ESMO). In order to improve successful implementation of PET imaging biomarkers into clinical practice, it is essential to identify potential hurdles. Recently, an international consensus meeting resulted in the “imaging biomarker roadmap,” describing the steps of imaging biomarkers towards clinical practice [1••]. In this review, we describe the current status of PET biomarkers for BC, according to this roadmap. We identify specific challenges for each tracer individually and make recommendations for next steps towards clinical implementation.

## Development Stages of Imaging Biomarkers

The imaging biomarker roadmap describes three parallel tracks, towards biomarker implementation in clinical practice [1••]. Technical validity, i.e., whether the test can be trusted, requires harmonization and standardization of techniques as an assessment of repeatability and reproducibility. Clinical validity, i.e., whether the test is clinically meaningful, addresses the discriminatory value to predict diagnosis, prognosis, or therapy response. Finally, clinical utility, i.e., whether the test improves patient outcome and is cost-effective, is determined by health-related measurements. Successful progress through these tracks is essential for a test to pass from analytical to clinical research stage, and subsequently to routine clinical practice [1••].

## Search Strategy

For this literature review, the database PubMed was searched until September 2019. PET tracers were included if Food and Drug Administration (FDA) approved or at least two prospective clinical articles, including ≥ 50 BC patients, were published within the past 5 years. As a result, four radiotracers were selected ([^18^F]-FDG, [^18^F]-NaF, 16α-[^18^F]-fluoroestradiol ([^18^F]-FES), and zirconium-89 [^89^Zr]-trastuzumab). Search terms were repeatability, reproducibility, inter- and intra-observer, diagnosis, prognosis, response to treatment, survival, metastases, technical and clinical validity/utility, cost-effectiveness, BC, PET, and meta-analysis.

## Development Stages of [^18^F]-FDG-PET/CT

### Technical Validity

[^18^F]-FDG-PET/computed tomography (CT) can detect increased glucose metabolism in cancer cells and is indicated for multiple oncological indications [2, 3]. [^18^F]-FDG is phosphorylated by the enzyme hexokinase and trapped inside (tumor) cells [4]. The reproducibility and repeatability of [^18^F]-FDG-PET/CT were assessed for various cancer types (see Table 1 for overview) [58]. One meta-analysis of 5 studies, including 102 cancer patients of which 6 had metastatic BC (MBC), assessed the repeatability of [^18^F]-FDG-PET(/CT) by measuring the standardized uptake value (SUV)_max/mean_ in the same patient on two separate occasions with an interval of 1–4 days [5]. A high test-retest interclass correlation coefficient (ICC) of 0.90 and 0.91 was found for SUV_max_ and SUV_mean_, respectively. Reproducibility across different scanners was assessed in 23 patients, 17 with BC [13]. Patients underwent two [^18^F]-FDG-PET/CT scans within 15 days on the same scanner or on different scanners at different sites. Cross-calibration of PET/CT scanners and dose calibrator was performed. The average difference in SUV_max_ between test-retest [^18^F]-FDG-PET/CT, using the same scanner, was 8% versus 18% on different scanners. International standardization efforts to improve reproducibility resulted in the European Association of Nuclear Medicine (EANM) guideline for ^18^F imaging procedures, followed in 2010 by the Research Ltd. (EARL) accreditation program to assure independent quality control, comparable scanner performance, and reproducible assessments [3, 59]. Since 2010, the number of accredited centers has increased over time in Europe and beyond [60].

**Table 1:** Development stages of [^18^F]-FDG-PET/CT, [^18^F]-NaF-PET/CT, [^18^F]-FES-PET/CT, and [^89^Zr]-trastuzumab PET/CT

Checklist	Article	No. of patients	Study type	Scanner	PET measurement	Reference standard	Level of evidence^¶^
Technical validity* [^18^F]-FDG-PET (FDA approved)							
Repeatability	Van Langen,2012 [5]	102, 5 studies	Meta-analysis (prospective/retrospective)	PET and PET/CT	Semi-quantitative (SUV)		II
Repeatability	Kramer, 2016 [6]	9	Prospective	PET/CT	Semi-quantitative (SUV, TLG, MATV)		III
Repeatability	Weber, 2015 [7]	74	Prospective	PET/CT	Semi-quantitative (SUV)		III
Repeatability	Rockall, 2016 [8]	21	Prospective	PET/CT	Semi-quantitative (SUV)		III
Repeatability	Fraum, 2019 [9]	14	Prospective	PET/CT	Semi-quantitative (SUV, SUL)		III
Repeatability	Frings, 2014 [10]	34	Prospective	PET/CT	Semi-quantitative (SUV)		III
Repeatability	Hoang, 2013 [11]	17	Prospective	PET/CT	Semi-quantitative ((Δ)SUV)		III
Repeatability	Van Velden, 2014 [12]	29	Prospective	PET/CT	Semi-quantitative (SUV, TLG)		III
Reproducibility	Kurland, 2019 [13]	23	Prospective	PET/CT	Semi-quantitative (SUV)		III
Reproducibility	Goh, 2012 [14]	25	Prospective	PET/CT	Semi-quantitative (SUV)		III
Repeatability/reproducibility	Heijmen, 2012 [15]	20	Prospective	PET/CT	Semi-quantitative (SUV, TLG, volume)		III
Repeatability/reproducibility	Kolinger, 2019 [16]	10	Prospective	PET/CT	Semi-quantitative (SUV)		III
Repeatability/reproducibility	Rasmussen, 2015 [17]	30	Prospective	PET/CT	Semi-quantitative (SUV, MTV, TLG)		III
Technical validity* [^18^F]-NaF-PET (FDA approved)							
Repeatability	Lin, 2016 [18]	35	Prospective	PET/CT	Semi-quantitative (SUV)		III
Repeatability	Wassberg, 2017 [19]	10	Prospective	PET/CT	Visual and semi-quantitative (SUV, FTV, TLF)		III
Repeatability	Kurdziel, 2012 [20]	Subgroup of 21	Prospective	PET/CT	Semi-quantitative (SUV)		III
Reproducibility	Zacho, 2019 [21]	219	Prospective	PET/CT	Visual		III
Technical validity* [^18^F]-FES-PET							
Reproducibility	Chae, 2019 [22••]	90	Prospective	PET/CT	Visual		III
Technical validity^†^ [^89^Zr]-trastuzumab-PET: no data are available							
Clinical validity^†^ [^18^F]-FDG-PET (FDA approved)							
Diagnosis - primary tumor	Bertagna, 2013 [23]	NR, 13 studies	Meta-analysis (prospective/retrospective)	PET and PET/CT	NR	Partly based on pathology	II
Diagnosis - primary tumor	Zhang, 2018 [24•]	2890, 39 studies	Meta-analysis (NR)	PET and PET/CT	NR	Pathology	II
Diagnosis - axillary nodes	Cooper, 2011 [25]	2591, 26 studies	Meta-analysis (prospective/ retrospective)	PET and PET/CT	Visual	SLNB, ALND	II
Diagnosis - axillary nodes	Liang, 2016 [26•]	1887, 21 studies	Meta-analysis (prospective/retrospective)	PET/CT	Semi-quantitative (SUV)	Fine needle aspiration biopsy, SLNB, ALND	II
Diagnosis - axillary nodes	Pritchard, 2012 [27]	325	Prospective	PET and PET/CT	Visual	SLNB, ALND	III
Diagnosis - recurrence	Xiao, 2016 [28]	1752, 26 studies	Meta-analysis (prospective/retrospective)	PET and PET/CT	Visual	Pathology, clinical or imaging	II
Diagnosis - metastases	Hong, 2013 [29]	748, 8 studies	Meta-analysis (prospective/retrospective)	PET/CT	Visual, semi-quantitative (not specified)	Pathology, clinical or imaging	II
Diagnosis - bone metastases	Rong, 2013 [30]	668, 7 studies	Meta-analysis (prospective/retrospective)	PET/CT	Visual, semi-quantitative (not specified)	Pathology, clinical, or imaging	II
Prognosis - clinicopathological	Groheux, 2011 [31]	131	Prospective	PET/CT	Semi-quantitative (SUV)	Pathology	III
Prognosis - survival	Diao, 2018 [32•]	3574, 15 studies	Meta-analysis (prospective/retrospective)	PET and PET/CT	Semi-quantitative (SUV)	Not specified	II
Prognosis - survival	Evangelista, 2017 [33]	275	Prospective	PET/CT	Visual, semi-quantitative (SUV)	Pathology or imaging	III
Prognosis - survival	Zhang, 2013 [34]	244	Prospective	PET/CT	Semi-quantitative (SUV)	Pathology, clinical, or imaging	III
Therapy response - neoadjuvant	Liu, 2015 [35]	382, 6 studies	Meta-analysis (prospective/retrospective)	PET/CT	Semi-quantitative (ΔSUV)	Pathology	II
Therapy response - neoadjuvant	Tian, 2017 [36•]	1119, 22 studies	Meta-analysis (prospective/retrospective)	PET/CT	Semi-quantitative (ΔSUV)	Pathology	II
Therapy response - neoadjuvant	Coudert, 2014 [37]	142	Randomized, prospective	PET/CT	Semi-quantitative (SUV)	Pathology	II
Clinical validity^†^ [^18^F]-NaF-PET (FDA approved)							
Diagnosis	Withofs, 2011 [38]	24	Prospective	PET/CT	Visual	MRI or CT	III
Diagnosis	Damle, 2013 [39]	72	Prospective	PET/CT	Visual	Pathology or imaging	III
Diagnosis	Liu, 2019 [40•]	Subgroup of 125 (3 studies)	Meta-analyses (prospective/retrospective)	PET/CT	Visual	Pathology, clinical, or imaging	II
Prognosis - survival	Peterson, 2018 [41]	28	Prospective	PET/CT	Semi-quantitative ((Δ)SUV)	Not specified	III
Therapy response	Azad, 2019 [42]	12	Prospective	PET/CT	Semi-quantitative ((Δ)metabolic flux, SUV)	Clinical or imaging	III
Therapy response	Azad, 2019 [43]	16	Prospective	PET/CT	Semi-quantitative ((Δ)SUV, TLM, MTV, SD, entropy, uniformity, kurtosis, skewness)	Clinical or imaging	III
Therapy response	Azad, 2019 [44]	22	Prospective	PET/CT	Semi-quantitative (ΔSUV)	Clinical or imaging	III
Clinical validity^†^ [^18^F]-FES-PET							
Diagnosis	Evangelista, 2016 [45]	238, 9 studies	Meta-analysis (prospective/retrospective)	PET and PET/CT	Semi-quantitative (SUV)	Partly based on pathology	II
Diagnosis	Chae, 2019 [22••]	90	Prospective	PET/CT	Visual and semi-quantitative (SUV)	Pathology	III
Diagnosis	Venema, 2017 [46]	13	Prospective	PET/CT	Semi-quantitative (SUV)	Pathology	III
Diagnosis	Gupta, 2017 [47]	10	Prospective	PET/CT	Visual and semi-quantitative (SUV)	Pathology	III
Prognosis	Kurland, 2017 [48]	90	Prospective	PET and PET/CT	Visual and semi-quantitative (SUV, SUL)	Clinical or imaging	III
Therapy response	Evangelista, 2016 [45]	183, 6 studies	Meta-analysis (prospective)	PET and PET/CT	Semi-quantitative (SUV)	Clinical or imaging	II
Therapy response	Chae, 2017 [49•]	26	Randomized, prospective	PET/CT	Semi-quantitative (SUV)	Pathology	II
Therapy response	Van Kruchten, 2015 [50]	19	Prospective	PET/CT	Semi-quantitative (SUV)	Clinical or imaging	III
Therapy response	Park, 2016 [51]	24	Prospective	PET/CT	Semi-quantitative (SUV)	Pathology, clinical, or imaging	III
Therapy response	Gong, 2017 [52]	22	Prospective	PET/CT	Semi-quantitative ((Δ)SUV)	Imaging	III
Clinical validity^†^ [^89^Zr]-trastuzumab-PET							
Diagnosis	Dehdashti, 2018 [53]	51	Prospective	PET/CT	Visual and semi-quantitative (SUV)	Pathology, clinical or imaging	III
Therapy response	Gebhart, 2016 [54]	56	Prospective	PET/CT	Visual and semi-quantitative (SUV)	[^18^F]-FDG-PET	III
Clinical utility^†^ [^18^F]-FDG-PET (FDA approved)							
Cost-effectiveness	Koleva-Kolarova, 2015 [55]	5073	Computer simulation	PET/CT	Costs and ICER		^§^
Clinical utility^†^ [^18^F]-NaF-PET (FDA approved): no data are available							
Clinical utility^†^ [^18^F]-FES-PET							
Cost-effectiveness	Koleva-Kolarova, 2015 [55]	5073	Computer simulation	PET/CT	Costs and ICER		^§^
Cost-effectiveness	Koleva-Kolarova, 2018 [56]	Hypothetical cohort of 1000	Computer simulation	PET/CT	Costs, LYG and ICER		^§^
Clinical utility^†^ [^89^Zr]-trastuzumab-PET							
Cost-effectiveness	Koleva-Kolarova, 2018 [56]	Hypothetical cohort of 1000	Computer simulation	PET/CT	Costs, LYG, and ICER		^§^

Articles are included if they met in- (prospective study design) and exclusion criteria (trials using PET only scanners or including less than 10 (breast cancer) patients (except clinical validity [^18^F]-FDG-PET ≥ 100 breast cancer patients)

*PET* positron emission tomography, *CT* computed tomography, *NR* not reported, *SUV* standardized uptake value, *TLG* total lesion glycolysis, *MATV* metabolic active tumor volume, *SUL* SUV normalized by lean body mass, *MTV* metabolic tumor volume, *TLM* total lesion metabolism, *FTV* functional tumor volume, *TLF* skeletal tumor burden, *SLNB* sentinel lymph node biopsy, *ALND* axillary lymph node dissection, *SD* standard deviation, *QALY* quality-adjusted life year, *ICER* incremental cost-effectiveness ratio, *LYG* life years gained

^¶^According to ESMO guidelines (I: large randomized trials of good methodological quality or meta-analyses of randomized trials, II: (small) randomized trials or meta-analyses of (small) trials, III: prospective studies, IV: retrospective studies, V: expert opinion) [57]

^§^Level of evidence does not fit the ESMO criteria

*The study can be performed in various solid tumors, not necessarily breast cancer. Repeatability: refers to measurements performed multiple times in the same subject using the same equipment, software and observers over a short timeframe. Reproducibility: refers to measurements performed using different equipment, different software or observers, or at different sites and times, either in the same or in different subjects

^†^Always performed in breast cancer patients

### Clinical Validity

For [^18^F]-FDG-PET/CT, we focused on clinical validity studies with at least 100 BC patients. A meta-analysis of 13 studies (see Table 1) reported incidental and unexpected breast uptake detected by [^18^F]-FDG-PET(/CT) [23]. Overlap between SUVs in malignant and benign breast incidentalomas was found, and not all lesions were further histologically examined. Therefore, [^18^F]-FDG-PET/CT is not routinely used for diagnosis of primary BC. With regard to diagnosis of axillary lymph node metastases in BC, a meta-analysis was performed of studies comparing [^18^F]-FDG-PET(/CT) to the reference standard: axillary lymph node dissection (ALND) or sentinel lymph node biopsy (SLNB) [25]. In 7 out of 26 studies involving 862 BC patients, [^18^F]-FDG-PET/CT sensitivity was 56% and specificity 96%, compared to 52% and 95% for ALND and/or SLNB [25]. Another meta-analysis (21 studies including 1887 BC patients), using ALND and/or SLNB as reference standard, showed a sensitivity and specificity of 64% and 93%, respectively, for detection of axillary lymph node metastases by [^18^F]-FDG-PET/CT [26•]. Based on these data, [^18^F]-FDG-PET/CT is not recommended in the EANM, NCCN, or ESMO guidelines for detection of axillary lymph node metastases. However, as axillary BC management has evolved over the last decades, the use of [^18^F]-FDG-PET/CT in this setting may change as well. For instance, according to the Dutch BC guideline, [^18^F]-FDG-PET/CT can be considered for staging of BC patients prior to neoadjuvant chemotherapy, although a biopsy of axillary lymph nodes with high [^18^F]-FDG uptake is advised to avoid false positive results [61]. With regard to [^18^F]-FDG-PET/CT for diagnosis of recurrent or distant metastases in BC, two meta-analyses including a total of 2500 patients (2 studies with overlapping subjects) showed both high sensitivity (92–96%) and specificity (82–95%) [28, 29]. For the detection of bone metastases, [^18^F]-FDG-PET/CT showed a sensitivity and specificity of 93% and 99%, versus 81% and 96% respectively, for conventional bone scintigraphy, as determined in a meta-analysis involving 668 BC patients in 7 studies [30]. According to the EANM, ESMO, and NCCN guidelines, [^18^F]-FDG-PET/CT should be considered in cases of suspected recurrence or equivocal findings on standard imaging and can be used for staging in high-risk BC patients [2, 3, 62, 63••, 64, 65••].

Despite the non-specific uptake of [^18^F]-FDG, preoperative [^18^F]-FDG uptake, expressed as SUV_max_, was found to be related to prognostic pathological characteristics assessed on core biopsy in primary BC. SUV_max_ was higher in ER− than ER+ tumors (7.6 versus 5.5); higher uptake was also observed in triple-negative tumors, tumor grade 3, ductal carcinoma, and p53 mutated tumors [31]. A meta-analysis of 15 studies with 3574 BC patients evaluated the prognostic value of [^18^F]-FDG uptake in primary breast lesions [32•]. High SUV_max_ was related to a higher risk of recurrence or progression compared with a low SUV_max_. However, the SUV_max_ cutoff values varied widely between studies, ranging from 3.0 to 11.1 [32•]. Lower baseline SUV_max_ predicted more favorable survival outcomes than higher SUV_max_ (analyzed as a continuous variable) [34]. The lack of clear cutoff values has so far precluded the use of [^18^F]-FDG-PET as a prognostic tool in BC. This is partly due to the fact that SUV calculations can depend on the PET camera systems used. To harmonize the acquisition protocols and the quantification process between different camera systems, the EARL harmonization program was introduced.

Clinical validity of serial [^18^F]-FDG-PET/CT to monitor therapy response to neoadjuvant treatment was analyzed in two meta-analyses (see Table 1), showing a pooled sensitivity of 82–86% and specificity of 72–79%, using histopathology as reference standard for pathological (non-)response [35, 36•]. Possibly differences between the pace of disease response between BC subtypes may play a role in this setting. In the randomized neoadjuvant study AVATAXHER in 142 patients with HER2+ BC, [^18^F]-FDG-PET/CT at baseline and after 1 cycle of docetaxel/trastuzumab was used for further treatment decisions [37]. Patients with a ΔSUV_max_ of ≥ 70% (*n* = 69) continued docetaxel/trastuzumab. Patients with a ΔSUV_max_ of < 70% (*n* = 73) were randomized for continued docetaxel/trastuzumab or addition of bevacizumab. In all patients receiving docetaxel/trastuzumab, this ΔSUV_max_ cutoff of 70% showed a positive and negative predictive value of 53% and 75%, respectively, to detect pathological complete response. Recently, preliminary data from the neoadjuvant PREDIX HER2 trial showed that pathological response was related to decreased uptake on early [^18^F]-FDG-PET/CT compared to baseline, in HER2+ primary BC [66]. For MBC, no well-designed large study to assess the clinical value of [^18^F]-FDG-PET/CT has been performed, only small studies with varying endpoints [67, 68]. The optimal cutoff value and interval between [^18^F]-FDG-PET/CT scans for response measurement in BC are still unknown and may limit implementation of [^18^F]-FDG-PET/CT as a tool for early response prediction in clinical practice. Attempts to integrate [^18^F]-FDG-PET/CT in the Response Evaluation Criteria in Solid Tumors (RECIST) criteria have not been successful so far, and [^18^F]-FDG-PET/CT is not routinely used for response evaluation in BC, due to the absence of sufficient clinical validation data [69••, 70].

### Clinical Utility

Evidence on the cost-effectiveness of [^18^F]-FDG-PET/CT in BC is limited (Table 1). A Dutch computer simulation study by Koleva-Kolarova et al. evaluated the effect of [^18^F]-FDG-PET/CT on the number of performed biopsies and additional costs compared to the standard clinical workup for diagnosing ER+ MBC patients, using the incremental cost-effectiveness ratio (ICER) to avoid a biopsy [55]. This study demonstrated a 38 ± 15% increase in biopsies, and higher costs for [^18^F]-FDG-PET/CT compared to standard workup.

## Conclusions and Recommendations of [^18^F]-FDG-PET/CT

**Fig. 1:**
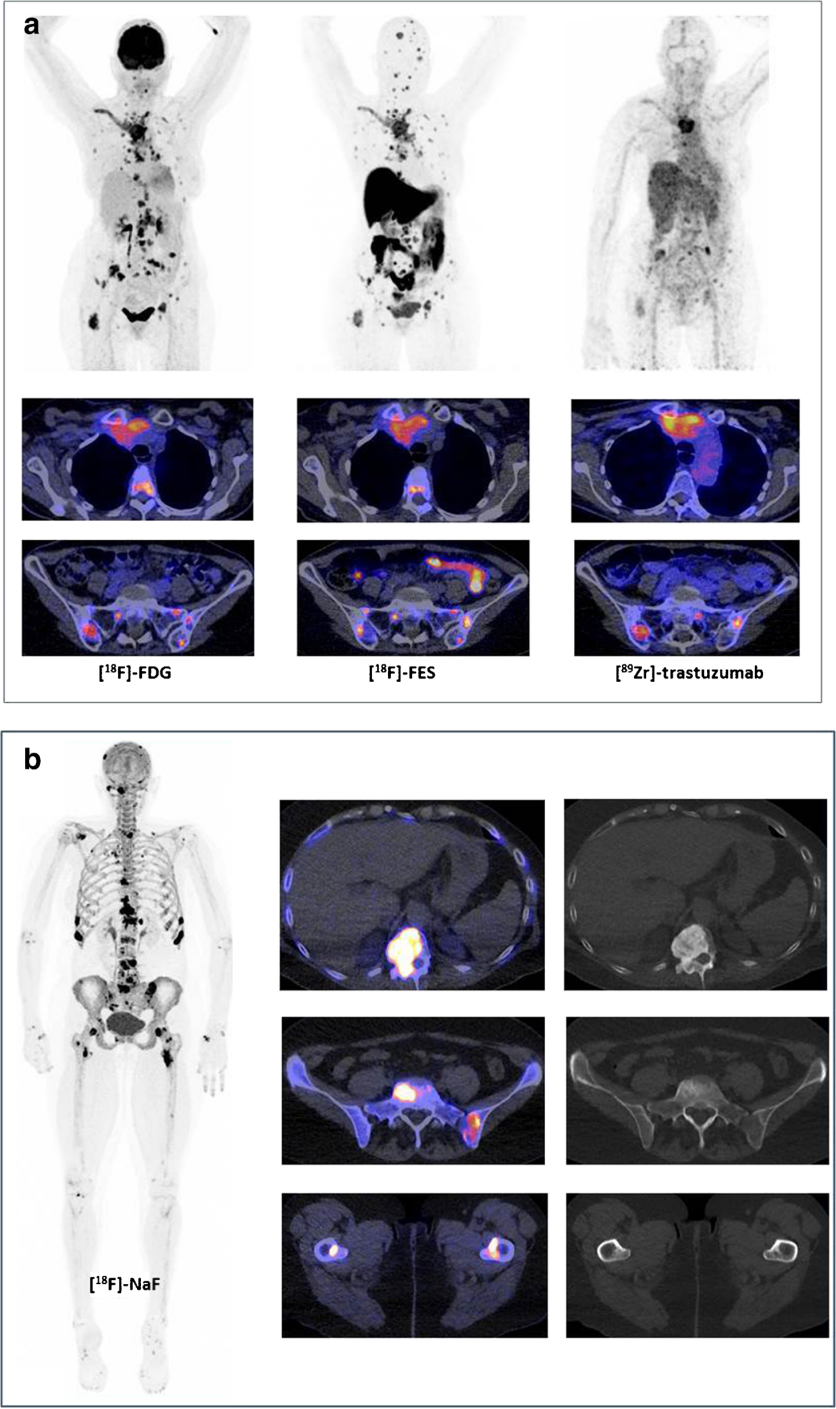
Upper image: three PET scans ([^18^F]-FDG-PET, [^18^F]-FES-PET, and [^89^Zr]-trastuzumab-PET) in the same patient showing mediastinal and hilar lymph node metastases, as well as intrapulmonary lesions visible on both [^18^F]-FDG-PET and [^18^F]-FES-PET, but not on [^89^Zr]-trastuzumab-PET. The large mediastinal mass (first row of transversal fused images) was visible on all three imaging modalities. Bone metastases (second row of transversal fused images) were clearly visualized on [^18^F]-FES-PET, for example, skull lesions, and to a lesser extent on [^18^F]-FDG-PET and [^89^Zr]-trastuzumab-PET. Lower image: [^18^F]-NaF-PET in another patient showing bone metastases in the skull, vertebrae, costae, pelvis, and proximal femora. The increased uptake in the joint was related to degeneration

While the technical validity track for [^18^F]-FDG-PET/CT has been completed successfully with international EARL and EANM standardization and harmonization of the technique itself, this harmonization is still lacking regarding clinical validity and utility. This has hampered routine use of [^18^F]-FDG-PET/CT in BC management worldwide. First, studies establishing a receiver operating characteristic (ROC) curve, sensitivity, and specificity in well-defined large cohort trials are needed, with biopsy as gold standard. The IMPACT breast trial (*NCT01957332*), in which baseline [^18^F]-FDG-PET/CT was performed in 200 MBC patients of all subtypes, including biopsy of a metastasis and conventional imaging, is likely to provide these data in the near future. Second, factors affecting [^18^F]-FDG-PET/CT results other than treatment effects should be standardized as much as possible (such as time of the scan after therapy). Finally, clinical utility assessment by integrating imaging biomarkers into randomized trials, developing an imaging data warehouse for EARL [^18^F]-FDG-PET/CT scans, and performing meta-analyses of these data may provide the final support for full implementation of [^18^F]-FDG-PET/CT into clinical practice (Fig. 1).

## Development Stages of [^18^F]-NaF-PET/CT

### Technical Validity

Bone is the most common site of metastasis in BC. Two PET tracers ([^18^F]-FDG and [^18^F]-NaF) are included in EANM and NCCN guidelines to identify bone metastases in BC patients. [^18^F]-NaF, approved by the FDA in 1972, reflects enhanced bone metabolism due to bone metastases but also due to degeneration, arthritis, or fractures [71, 72]. The repeatability of [^18^F]-NaF-PET/CT was evaluated in a prospective multicenter study by Lin et al. in 35 prostate cancer patients with bone metastases who underwent two pretreatment [^18^F]-NaF-PET/CT scans (test-retest interval 3 ± 2 days), with SUV_mean_ as most repeatable endpoint (overview: Table 1) [18]. Repeatability of SUV_mean/max_, functional tumor volume (FTV_50%_), and total lesion [^18^F]-fluoride uptake (TLF) measured with [^18^F]-NaF-PET/CT was confirmed by Wassberg et al. [19]. Moreover, a high inter-observer agreement at the patient level was found by using three scales to define [^18^F]-NaF-PET/CT findings [21]. How to correctly perform and interpret [^18^F]-NaF-PET/CT scans is published in EANM and Society of Nuclear Medicine and Molecular Imaging (SNMMI) guidelines, supporting technical standardization and harmonization [73, 74].

### Clinical Validity

At present, no comparison has been performed of [^18^F]-NaF-PET/CT with a bone biopsy as the gold standard for the entire study population, but it has been compared with other imaging modalities. [^18^F]-NaF-PET/CT has a higher sensitivity to detect bone metastases than either [^18^F]-FDG-PET/CT or conventional bone scintigraphy with ^99m^Tc-labeled diphosphonates (planar and SPECT) (97–100% versus 74% versus 91%, respectively). However, although the specificity of [^18^F]-NaF-PET/CT was higher than that of bone scintigraphy, it was slightly lower than [^18^F]-FDG-PET/CT (71–85% versus 63% and 97%, respectively) [39, 40•]. In general, a negative [^18^F]-NaF-PET/CT can be used to exclude bone metastases, but in case of positive findings, [^18^F]-NaF-PET/CT should be carefully interpreted and correlated with CT findings. With regard to the prognostic value of [^18^F]-NaF-PET/CT, one prospective study was performed in 28 BC patients with bone-dominant disease, showing no correlation between baseline SUV_max_ and skeletal-related events, time-to-progression or overall survival (OS) [41]. However, ΔSUV_max_ of 5 lesions between baseline and ~ 4 months of systemic treatment was associated with OS [41]. With regard to the predictive value of [^18^F]-NaF-PET/CT, two small studies showed that lack of endocrine treatment efficacy was related to an increase in metabolic flux to mineral bone or SUV_max_ in BC patients with bone only disease (see Table 1) [42, 44]. The national prospective oncologic PET registry of the USA showed that [^18^F]-NaF-PET/CT altered the treatment plan in 39% of BC patients [75]. However, the impact of [^18^F]-NaF-PET/CT for therapy response on clinical decision-making remains unclear due to varying endpoints and experimental procedures.

### Clinical Utility

The cost-effectiveness of [^18^F]-NaF-PET(/CT) to detect bone metastases was assessed in a meta-analysis of 11 trials, including 425 patients (7 BC patients) [76]. It was concluded that the average cost-effective ratio was less favorable for [^18^F]-NaF-PET(/CT) than for conventional bone scintigraphy.

## Conclusions and Recommendations of [^18^F]-NaF-PET/CT

While the technical validation of [^18^F]-NaF-PET/CT is completed, clinical validation with comparison to a biopsy as reference standard is still warranted. Also, clinical validity of [^18^F]-NaF-PET/CT should be further assessed with uniform endpoints. Therefore, [^18^F]-NaF-PET/CT has not yet passed through the necessary steps towards routine clinical practice according to the imaging biomarker roadmap. Although in bone-trope cancers such as BC, an optimal tool for diagnosis and treatment evaluation is still needed and it is unclear whether this tool could be [^18^F]-NaF-PET/CT.

## Development Stages of [^18^F]-FES-PET/CT

### Technical Validity

[^18^F]-FES-PET/CT enables the visualization of ER expression, with [^18^F]-FES behaving very similar to estradiol [77]. A large prospective cohort study of 90 BC patients with first recurrence/metastatic disease and preliminary results from a prospective study in 10 ER+ MBC patients showed an excellent inter-observer agreement for [^18^F]-FES uptake (0.90 and 0.98, respectively) [22••, 78]. Although limited data about repeatability and reproducibility are available, a recent guideline paper does provide recommendations regarding standardization of scanning time, control of pre-analytical factors that influence [^18^F]-FES uptake (such as discontinuation of estrogen receptor degraders > 5 weeks prior to scanning), visual analysis, and quantification of [^18^F]-FES uptake [77].

### Clinical Validity

A meta-analysis of 9 studies (all prospective, except one) involving 238 patients reported a pooled sensitivity of 82% and specificity of 95% to detect ER+ tumor lesions by quantitative assessment of [^18^F]-FES uptake (overview: Table 1) [45]. A similar sensitivity and specificity was found in direct comparison of [^18^F]-FES uptake and ER expression on biopsy (in 5 studies including 158 BC patients) [45]. Recently, a large prospective cohort study was published involving 90 BC patients with first recurrence/metastatic disease, comparing the correlation between qualitative [^18^F]-FES-PET/CT results and immunohistochemistry (IHC) of ER status of the same metastatic lesion. This resulted in a positive and negative predictive value of 100% and 78%, respectively [22••]. A quantitative analysis was also performed, showing a positive and negative agreement of [^18^F]-FES-PET/CT (threshold SUV_max_ 1.5) with ER IHC equaling 85% and 79%, respectively. Despite the importance of this well-defined prospective cohort trial, its impact is likely limited due to exclusion of bone metastases, the most common metastatic site in ER+ MBC. Furthermore, an optimal SUV_max_ cutoff to distinguish benign from malignant lesions by [^18^F]-FES-PET/CT has not been established. Although SUV_max_ 1.5 is most commonly used for this distinction, ranges of 1.0 to 2.0 have also been described. Yang et al. determined an ROC curve in 46 ER+ BC patients, showing an optimal SUV_max_ cutoff of 1.8, with a sensitivity of 88% and specificity of 88% (optimal SUV_mean_ cutoff: 1.2) [79]. The study of Nienhuis et al. in 91 ER+ MBC patients found that physiological background uptake could exceed SUV_max_ 1.5, for example, in the lumbar spine [80]. [^18^F]-FES-PET/CT scans performed in 108 individuals showed that irradiation could induce atypical (non-malignant) enhanced [^18^F]-FES uptake in the lungs [81]. These issues should be taken into account in interpreting [^18^F]-FES-PET/CT scans for the diagnosis of BC. However, these data are retrospective and should be interpreted with caution. Nonetheless, two trials have indicated usefulness of [^18^F]-FES-PET(/CT) for the physician by improving diagnostic understanding compared to conventional assessments in 88% of patients, and causing a treatment change in 48–49% of patients enrolled in the studies [82, 83]. Therefore, [^18^F]-FES-PET/CT may be a useful diagnostic tool in exceptional diagnostic dilemmas when added to a conventional workup. A prospective study involving 90 ER+ BC patients treated with endocrine therapy found that [^18^F]-FES-PET(/CT) may be a useful prognostic biomarker for [^18^F]-FDG avid tumors, demonstrating a higher median progression-free survival (PFS) in the high [^18^F]-FES uptake group compared to low [^18^F]-FES uptake group (7.9 versus 3.3 months, respectively) [48]. With regard to response prediction, a meta-analysis including 6 prospective trials and 183 patients found a pooled sensitivity of 64% and specificity of 29% to predict early or late response to hormonal therapy, with an SUV_max_ cutoff of 1.5, and a sensitivity of 67% and specificity of 62% with SUV_max_ of 2.0 [45]. In 26 patients with primary ER+ BC, randomized to neoadjuvant chemotherapy or endocrine treatment, no differences in baseline SUV_max_ were found between post-treatment pathological (non-) responders [49•]. In another small trial (including 18 patients), pathological response to neoadjuvant chemotherapy was related to low rather than high baseline SUV_max_ (1.8 versus 4.4) [84]. Overall, it is difficult to compare this data due to the heterogeneity of the trials, i.e., different endpoints, and imaging procedures.

### Clinical Utility

Two computer simulation studies described the impact of [^18^F]-FES-PET/CT on health-related measurements, such as life years gained (LYG), ICER, and total costs (Table 1) [55, 56]. One study selected first-line treatment in MBC patients based on biopsy results or [^18^F]-FES-PET/CT imaging findings and showed higher diagnostic and treatment costs in the PET/CT imaging group [56]. A second study determined the number of avoided biopsies to assess MBC after the introduction of [^18^F]-FES-PET/CT and showed that the number of biopsies (39 ± 9%) was lower in the [^18^F]-FES-PET/CT imaging group [55].

## Conclusions and Recommendations of [^18^F]-FES-PET/CT

While [^18^F]-FES-PET/CT is currently used in a limited number of hospitals worldwide, mostly in a research setting, but also as a diagnostic tool in exceptional diagnostic dilemmas, consistent data to support its clinical validity and utility are still lacking. Only in France is [^18^F]-FES approved for routine clinical use to determine ER status in MBC. In order to implement [^18^F]-FES-PET/CT more broadly in routine clinical practice, additional studies are needed. Within two prospective cohort trials, the multicenter IMPACT breast trial and the ECOG-ACRIN trial (*NCT02398773*; 99 newly diagnosed MBC patients), the analysis of baseline [^18^F]-FES uptake related to treatment response or PFS is ongoing. In the ongoing ET-FES TRANSCAN trial (*EUDRACT 2013-000-287-29*), the treatment choice is based on [^18^F]-FES-PET/CT (high versus low ^18^F-FES uptake) [85]. [^18^F]-FES-PET/CT is also added as integrated biomarker to another randomized controlled trial, the SONI*mage* trial (*NCT04125277*). With these additional studies, sufficient evidence could potentially be generated to support implementation of [^18^F]-FES-PET/CT in routine clinical practice.

## Development Stages of [^89^Zr]-Trastuzumab-PET/CT

### Technical Validity

The [^89^Zr]-labeled antibody trastuzumab binds to the HER2-receptor and has a relatively long half-life (t ½ = 78 h). This enables imaging at late time points but also limits repeatability testing as radiation dose is high and repeated scans would require a 2-week interval [86]. To optimize the acquisition protocol, imaging at multiple time points (after 1–7 days) was performed after a single tracer injection [87, 88]. The optimal time point was found after 4–5 days, due to lower background uptake and higher contrast. Recently, a [^89^Zr]-PET/CT EARL accreditation program was established, similar to [^18^F]-FDG-PET/CT accreditation [60, 89, 90••].

### Clinical Validity

No comparison of [^89^Zr]-trastuzumab-PET/CT with biopsy has been performed so far. In a prospective study including 34 HER2+ and 16 HER2− BC patients, an SUV_max_ cutoff of 3.2 showed a sensitivity of 76% and specificity of 62% to distinguish HER2+ from HER2− lesions [53]. The HER2 status was based on the primary tumor or metastatic lesion; however, a recent biopsy of a tumor lesion was not performed in all patients. Despite this relatively low discriminative value, [^89^Zr]-trastuzumab-PET/CT did support diagnostic understanding and resulted in a treatment change in 90% and 40% of patients respectively, in whom HER2 status could not be determined by standard workup [91]. With regard to the prognostic value of [^89^Zr]-trastuzumab-PET/CT no data are available, but its value to predict therapy response was assessed in the ZEPHIR trial (see Table 1) [54]. In 56 HER2+ MBC patients, qualitative analysis of baseline PET/CT scans indicated that [^89^Zr]-trastuzumab uptake was related to longer trastuzumab emtansine treatment duration, compared to no uptake (11.2 versus 3.5 months) [54].

### Clinical Utility

A computer simulated study of a hypothetical cohort of 1000 MBC patients assessed whether [^89^Zr]-trastuzumab-PET/CT could replace biopsy [56]. This study concluded that total costs were higher with [^89^Zr]-trastuzumab-PET/CT. However, biopsy effects on quality of life were not included in the analysis.

## Conclusions and Recommendations of [^89^Zr]-Trastuzumab-PET/CT

Although technical standardization and harmonization is supported by the recently introduced [^89^Zr]-PET/CT EARL accreditation program, at present, still significant knowledge gaps exist (for instance regarding the relation between biopsy and uptake on [^89^Zr]-trastuzumab-PET/CT) [89]. Therefore, multiple steps according to the imaging biomarker roadmap have to be taken before [^89^Zr]-trastuzumab-PET/CT can be implemented in clinical practice. It is expected that the previously mentioned multicenter IMPACT breast study will provide information that can advance the validation of [^89^Zr]-trastuzumab-PET/CT.

## Other PET Tracers for Molecular Imaging in BC

Multiple new tracers of potential interest in BC can be identified (see Table 2). PET imaging of additional receptors may be the next step, for example, the hormone receptor tracer [^18^F]-dihydrotesterone ([^18^F]-FDHT)-PET, which is commonly used in prostate cancer trials. This tracer provides information about androgen receptor (AR) expression, which is a potential new target for BC treatment [46]. Moreover, cell proliferation can be detected by [^18^F]-fluorothymidine ([^18^F]-FLT)-PET, and post-neoadjuvant chemotherapy [^18^F]-FLT uptake may be correlated with the proliferation marker Ki-67 measured by IHC in primary BC patients [92]. In light of the current developments in BC immunotherapy, assessment of the programmed death-ligand 1 (PD-L1) with [^89^Zr]-labeled atezolizumab is clearly of interest. Recently, a first-in-human study with 22 patients (including 4 with triple-negative BC) showed a better correlation of [^89^Zr]-atezolizumab uptake to treatment response, PFS and OS at patient level than the commonly used SP142 IHC marker [93]. Currently, one recruiting [^89^Zr]-atezolizumab-PET study is available for lobular BC (*NCT04222426*). Furthermore, a combination of molecular imaging techniques, such as [^18^F]-FES-PET, [^89^Zr]-trastuzumab-PET with [^18^F]-FDG-PET, may be useful in identifying disease heterogeneity or differentiating between indolent and aggressive disease [48, 54, 94]. This could help to select the best therapeutic strategy.

**Table 2:** Ongoing PET imaging based clinical trials including breast cancer patients (*n* = 48)

Radiotracer	Target	Description of disease characteristics	Estimated enrollment	Phase	Trial ID	(estimated) Study start year	Status
[^18^F]-FES	ER	ER+, HER2− MBC	60	NA	NCT03442504	2017	Recruiting
		ER+, HER2− MBC	8	I/II	NCT04150731	2020	Not yet recruiting
		ER+ (M)BC	60	III	NCT03544762	2017	Recruiting
		ER+, HER2− MBC	75	II	NCT02409316	2015	Recruiting
		ER+ MBC	68	NA	NCT03768479	2017	Recruiting
		ER+, HER2− MBC	104	I	NCT03455270	2018	Recruiting
		ER+, HER2− locally advanced and locoregional recurrent BC	40	NA	NCT03726931	2018	Recruiting
		ER−, HER2+ MBC	33	NA	NCT03619044	2019	Not yet recruiting
		ER+ MBC	100	NA	NCT04125277	2019	Recruiting
		ER+ MBC	99	II	NCT02398773	2016	Recruiting
		ER+, HER2− MBC	25	NA	NCT03873428	2020	Not yet recruiting
		ER+ (M)BC	100	I	NCT01916122	2013	Recruiting
		ER+ recurrent BC or MBC	100	NA	NCT00816582	2010	Active, not recruiting
		Regardless of ER/HER2 status, MBC	217	NA	NCT01957332	2013	Active, not recruiting
		ER+ (M)BC	29	NA	NCT02149173	2010	Active, not recruiting
		ER+, HER2− MBC	16	I	NCT02650817	2016	Active, not recruiting
		ER+ MBC	15	NA	NCT01720602	2012	Active, not recruiting
[^18^F]-FDHT	AR	AR+, HER2− MBC	22	II	NCT02697032	2016	Active, not recruiting
[^18^F]-FTT	PARP-1	(M)BC	30	NA	NCT03846167	2019	Recruiting
		BC	30	I	NCT03083288	2017	Active, not recruiting
[^18^F]-ISO-1	Sigma-2 receptor	MBC	30	NA	NCT03057743	2016	Recruiting
		BC	30	I	NCT02284919	2014	Active, not recruiting
[^18^F]-FLT	Thymidine kinase activity	Regardless of ER/HER2 status, Rb + MBC	20	I	NCT02608216	2015	Recruiting
		MBC	17	NA	NCT01621906	2012	Active, not recruiting
[^18^F]-FMISO	Hypoxic cells	ER−, HER2− MBC	126	II	NCT02498613	2016	Recruiting
[^18^F]-GE-226	HER2	MBC	16	NA	NCT03827317	2019	Recruiting
[^18^F]-F-GLN	Glutamine metabolism	(M)BC	30	NA	NCT03863457	2019	Recruiting
[^18^F]-αvβ6-BP	α_v_β_6_	(M)BC	27	I	NCT03164486	2016	Recruiting
[^18^F]-Var3	Extracellular pH	MBC	10	I	NCT04054986	2019	Recruiting
[^18^F]-Flutemetamol	Amyloid beta	BC	15	NA	NCT02317783	2015	Recruiting
[^18^F]-FSPG	Amino acid transporter x_c_^−^	BC	120	NA	NCT03144622	2016	Recruiting
[^18^F]-FAZA	Hypoxic cells	BC	25	I	NCT03168737	2017	Recruiting
[^18^F]-ASIS	Tissue factor	(M)BC	10	I	NCT03790423	2019	Recruiting
[^89^Zr]-Trastuzumab	HER2	Regardless of ER/HER2 status, MBC	217	NA	NCT01957332	2013	Active, not recruiting
[^89^Zr]-Atezolizumab	PD-L1	ER−, HER2− MBC	54	NA	NCT02453984	2016	Recruiting
		Lobular ER+ MBC	10	NA	NCT04222426	2019	Recruiting
[^89^Zr]-CED88004S	CD8	ER−, HER2− MBC	40	I/II	NCT04029181	2019	Recruiting
[^89^Zr]-Bevacizumab	VEGF	Inflammatory HER2− (M)BC	10	I	NCT01894451	2015	Active, not recruiting
[^68^Ga]-ABY-025	HER2	HER2+ (M)BC	120	NA	NCT03655353	2018	Recruiting
[^68^Ga]-RM2	Gastrin-releasing peptide receptor	ER+ BC	80	III	NCT03731026	2018	Not yet recruiting
[^68^Ga]-NOTA-Anti-HER2 VHH1	HER2	MBC	20	II	NCT03924466	2019	Recruiting
		MBC	30	II	NCT03331601	2017	Recruiting
[^68^Ga]-FAPI-46	Fibroblast activated protein	(M)BC	30	I	NCT04147494	2019	Not yet recruiting
[^68^Ga]-PSMA-11	Prostate specific membrane antigen	(M)BC	30	I	NCT04147494	2019	Not yet recruiting
[^64^Cu]-DOTA-Trastuzumab	HER2	HER2+ BC	20	II	NCT02827877	2016	Recruiting
		HER2+ MBC	18	NA	NCT01093612	2011	Active, not recruiting
		HER2+ MBC	10	NA	NCT02226276	2015	Active, not recruiting
[^64^Cu]-DOTA-alendronate	Mammary microcalcifications	BC	6	I	NCT03542695	2020	Not yet recruiting
[^64^Cu]-M5A	Carcinoembryonic antigen	(M)BC	20	NA	NCT02293954	2015	Active, not recruiting
[^13^N]-NH3	Glutamine synthetase	Locally advanced BC	124	II	NCT02086578	2014	Active, not recruiting

Searched for breast cancer and positron emission tomography in ClinicalTrials.gov. Only trials which have not been published and had a recruitment status of active, (not yet) recruiting were included. Combined PET**/**MRI scans and [^**18**^F]-FDG-PET scans were excluded

*(M)BC* (metastatic) breast cancer, *ER* estrogen receptor, *HER2* human epidermal growth factor receptor 2, *AR* androgen receptor, *NA* not applicable, *PARP* poly ADP ribose polymerase, *PD-L1* programmed death-ligand 1, *VEGF* vascular endothelial growth factor

## Conclusions

In this review, we identified hurdles based on the biomarker roadmap for the four most commonly used PET tracers in BC and made recommendations for the next steps towards clinical implementation. This review has summarized several important steps to be considered to successfully implement molecular biomarkers for BC patients in clinical practice. In general, support for clinical utility is still pending for PET tracers in BC, but also assessment of clinical validity is hampered by varying endpoints and procedures. Improving trial designs can contribute to solve this matter; for instance, multicenter trials require standardization and harmonization of procedures. International collaboration is essential, as this would also potentially allow building warehouses of data to overcome a plethora of small solitary single center studies. Based on these warehouses, clinical validation can be established in line with the RECIST guidelines. In this setting, considering all aspects of the biomarker roadmap at an early stage is important. Smart trial designs adding imaging biomarkers to randomized controlled trials (integrated biomarker) are desirable, as imaging biomarker–based randomized controlled trials (integral biomarker) are usually not feasible due to the large numbers of patients required [95]. From a regulatory point of view, the evidence required for implementation is still unclear, although European Medicines Agency and FDA acknowledge that a microdose radiopharmaceutical is not similar to a therapeutic drug in this respect [96, 97]. Nonetheless, establishing whether patient outcome is truly improved is essential to justify implementation of a complex, expensive tool with radiolabeled PET tracers. A considerable international, collaborative effort could potentially make this possible.
